# Supplementary material for: Signatures of quantum spin liquid state and unconventional transport in thin film TbInO3
Source: Nat Commun. 2025 Oct 27;16:9469. doi: 10.1038/s41467-025-64528-2 (PMC12559177; doi:10.1038/s41467-025-64528-2)
Supplement: Supplementary file 1 — Supplementary Information [file 41467_2025_64528_MOESM1_ESM.pdf]

Supplement For
Signatures of Quantum Spin Liquid State and Unconventional Transport in Thin Film $TbInO_3$

Johanna Nordlander,[1,2,3,14,*] Margaret A. Anderson,[1,14] Tony Chiang,[4] Austin Kaczmarek,[5] Nabaraj Pokhrel,[6] Kuntal Talit,[7] Spencer Doyle,[1] Edward Mercer,[8,9] Christian Tzschaschel,[10] Jun-Ho Son,[11] Hesham El-Sherif,[12] Charles M. Brooks,[1] Eun-Ah Kim,[11] Alberto de la Torre,[8,9] Ismail El Baggari,[12] Elizabeth A. Nowadnick,[7] Katja C. Nowack,[5] John T. Heron,[4] and Julia A. Mundy[1,13,*]

[1] *Department of Physics, Harvard University, Cambridge, MA 02138, USA*

[2] *Paul Drude Institute for Solid State Electronics, 10117 Berlin, Germany*

[3] *Department of Physics, University of Zurich, 8057 Zurich, Switzerland*

[4] *Department of Materials Science and Engineering, University of Michigan, Ann Arbor, MI 48109, USA*

[5] *Department of Physics, Laboratory of Atomic and Solid State Physics, Cornell University, Ithaca, New York 14853, USA*

[6] *Department of Physics, University of California, Merced, CA 95343, USA*

[7] *Department of Chemical and Materials Engineering, University of California, Merced, CA 95343, USA*

[8] *Department of Physics, Northeastern University, Boston, MA 02115, USA*

[9] *Quantum Materials and Sensing Institute, Northeastern University, Burlington, MA 01803, USA*

[10] *Department of Chemistry and Chemical Biology, Harvard University, Cambridge, MA 02138, USA*

[11] *Department of Physics, Cornell University, Ithaca, NY 14853, USA*

[12] *Rowland Institute, Harvard University, Cambridge, MA 02138 USA*

[13] *School of Engineering and Applied Sciences, Harvard University, Cambridge, MA 02138, USA*

[14] *These authors contributed equally: Johanna Nordlander, Margaret A. Anderson.*

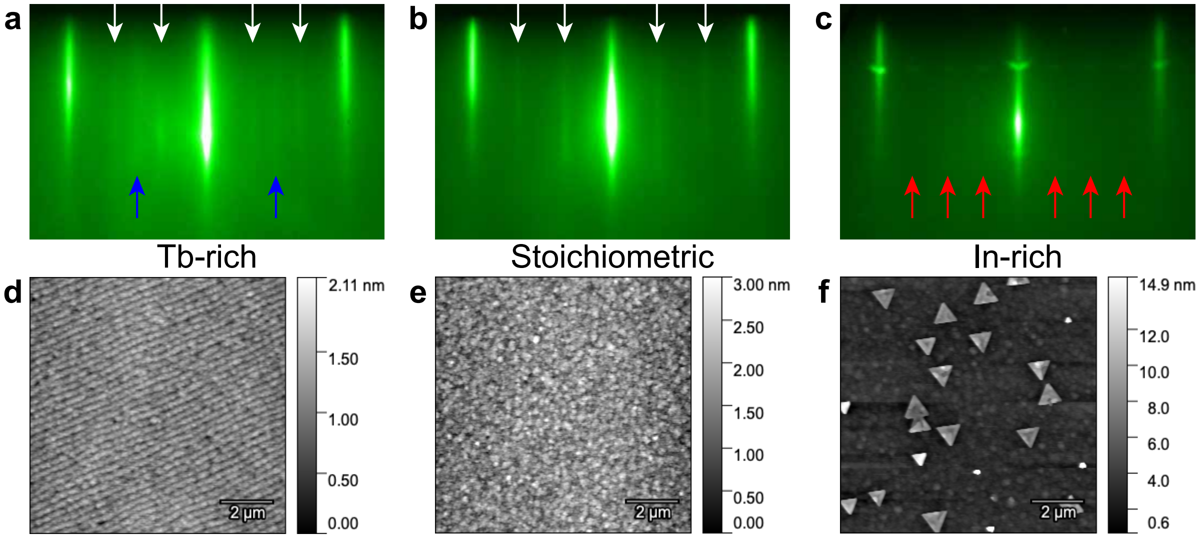

FIG. S1. Characterization of $TbInO_3$ thin films with varying stoichiometry. (a-c) In situ reflection high energy electron diffraction (RHEED) images taken along [100] after $TbInO_3$ thin-film growth on YSZ(111) for different growth conditions, from Tb-rich (a), through stoichiometric (b), to In-rich (c) conditions. The ferroelectric lattice trimerization is seen as a tripling of the unit cell and indicated by the additional streaks highlighted with white arrows on the top of the images. Impurity phases form in both Tb-rich and In-rich conditions, indicated by blue and red arrows at the bottom of the images. (d-f) Atomic force microscopy (AFM) images of Tb-rich (d), stoichiometric (e), and In-rich (f) $TbInO_3$ films. In-rich films are identified by the presence of triangular In_xO_y islands (see Fig. S2).

* E-mail: johanna.nordlander@physik.uzh.ch; mundy@fas.harvard.edu

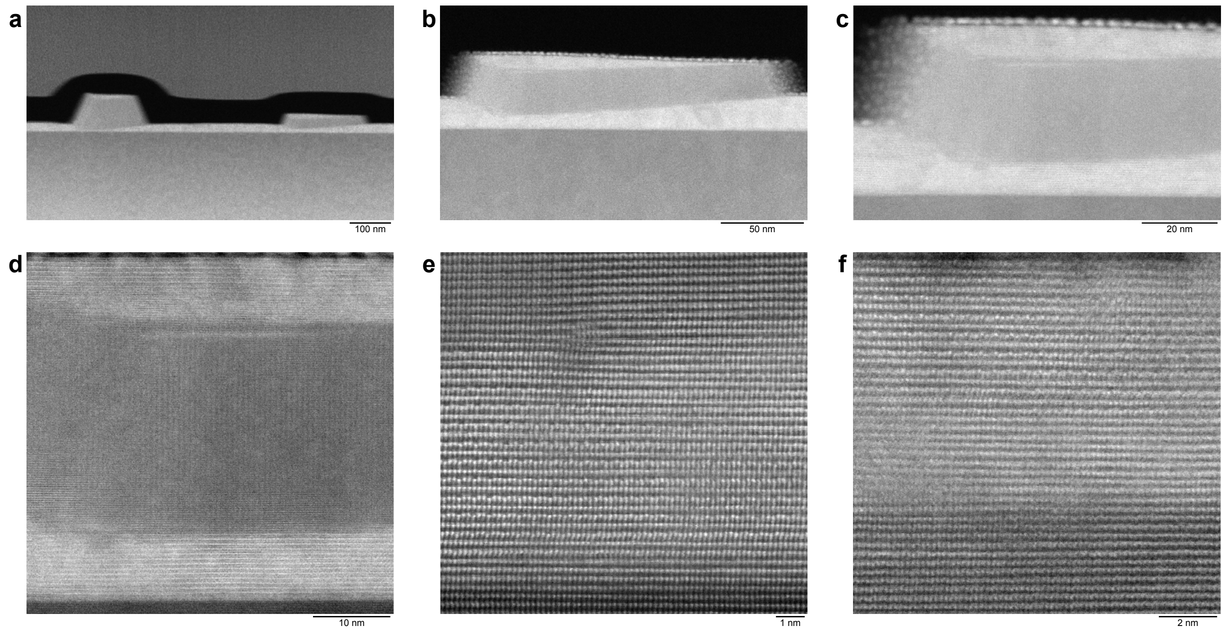

FIG. S2. HAADF-STEM imaging of In_xO_y islands in an In-rich $TbInO_3$ thin film on YSZ(111). (a) A wide field-of-view with two In_xO_y islands with varying morphology embedded in a $TbInO_3$ film. The islands are covered with a layer of sputtered carbon and e-beam deposited platinum to protect the film during lamella preparation. (b) A closer view of the right island in (a). Atop the YSZ(111) substrate, the $TbInO_3$ film has the brightest contrast with the slightly darker In_xO_y embedded within the top of the film. Additional $TbInO_3$ has grown atop the In_xO_y island. The rough particles on the surface of the island and film are sputtered platinum deposited to protect the film during lamella preparation. (c) A closer view of the island in (b). (d) With higher magnification, the layered structure of $TbInO_3$ is visible both below (in the film) and above (atop the island) of the embedded In_xO_y. (e) Atomic resolution HAADF-STEM shows the trimer distortion in the terbium sublattice of the $TbInO_3$ film beneath the incipient In_xO_y. (f) The $TbInO_3$ atop the In_xO_y island also hosts the signature improper ferroelectric polarization.

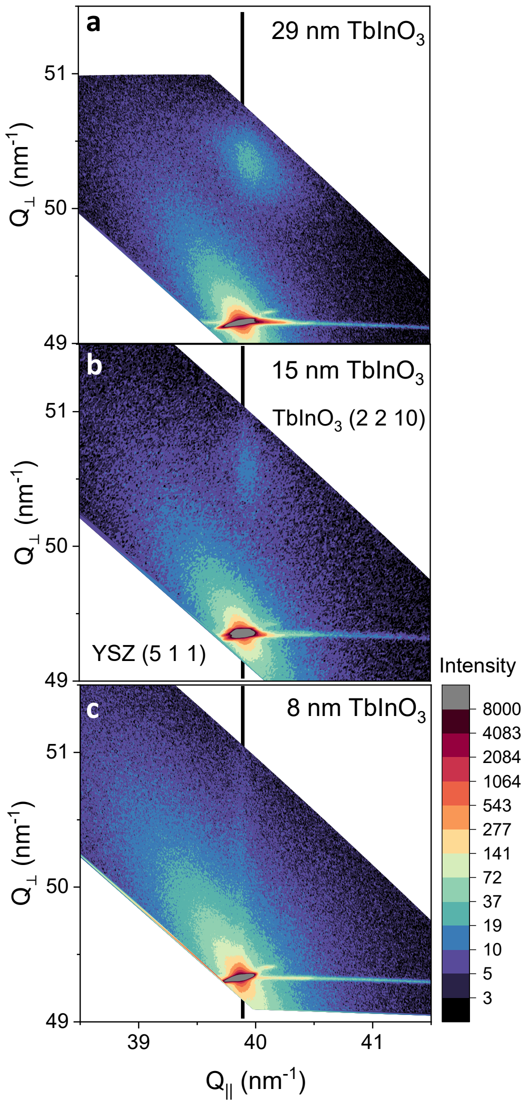

FIG. S3. X-ray reciprocal space mapping around the (2 2 10) reflection of $TbInO_3$ for different film thicknesses: (a) 29 nm, (b) 15 nm and (c) 8 nm. While the 8 nm film shows a film peak that is fully aligned with the substrate peak as indicated by the vertical black line, and hence fully strained, the 15 nm and 29 nm film peaks are slightly shifted, signifying partial relaxation.

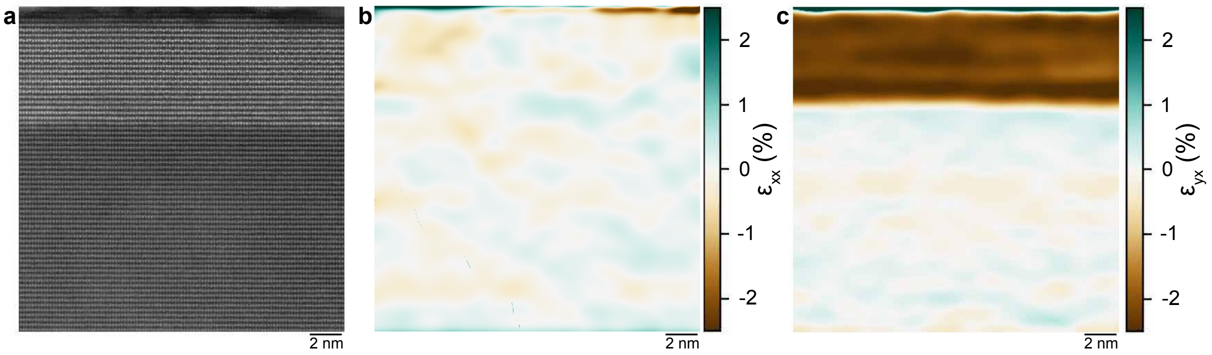

FIG. S4. Strain mapping in an 8 nm TbInO3 on YSZ(111). (a) A HAADF-STEM micrograph of the 8 nm thick TbInO3 film (top) and YSZ substrate (bottom) and a transitional layer with a cubic structure in between. (b-c) Strain maps of ϵ_{xx} and ϵ_{yx} in the region shown in (a) derived from a lock-in phase analysis analogous to geometric phase analysis. Uniform ϵ_{xx} across the interface (b) suggests the film is fully strained to the substrate. Finite ϵ_{yx} clearly identifies regions with the ideal hexagonal $TbInO_3$ structure. The cubic transition layer is identified as the interface region with similar STEM contrast as the $TbInO_3$ film in (a) yet with $\epsilon_{yx} \sim 0$ in (c), and corresponds here to the first four atomic layers away from the substrate interface. The cubic phase of this transitional layer is likely seeded by the cubic symmetry of the substrate.

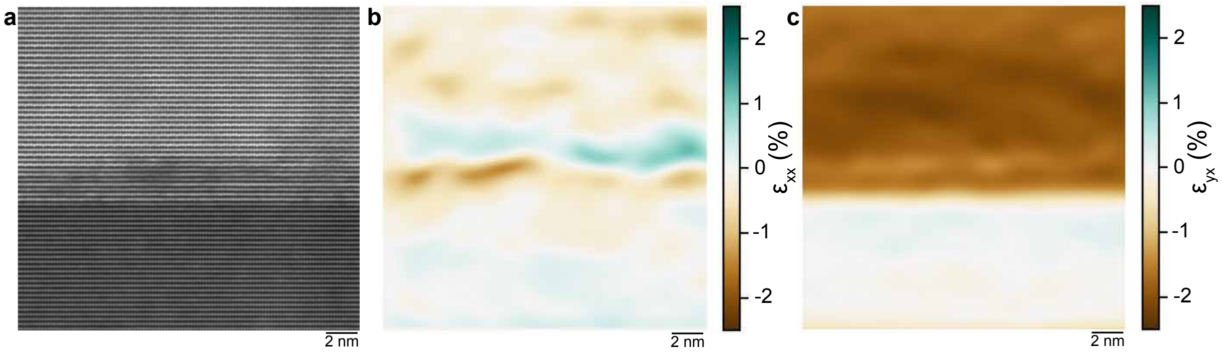

FIG. S5. Strain mapping in 15 nm $TbInO_3$ on YSZ(111). (a) A HAADF-STEM micrograph of the interface between the $TbInO_3$ film (top) and YSZ(111) substrate (bottom) with a cubic transitional region of about three atomic layers at the interface determined in the same way as in Fig. S4. (b-c) Strain maps derived from a lock-in phase analysis showing increased ϵ_{xx} at the interface and small negative ϵ_{xx} within the film (b), which indicates partial relaxation of the film, and homogeneous, finite ϵ_{yx} within the film (c).

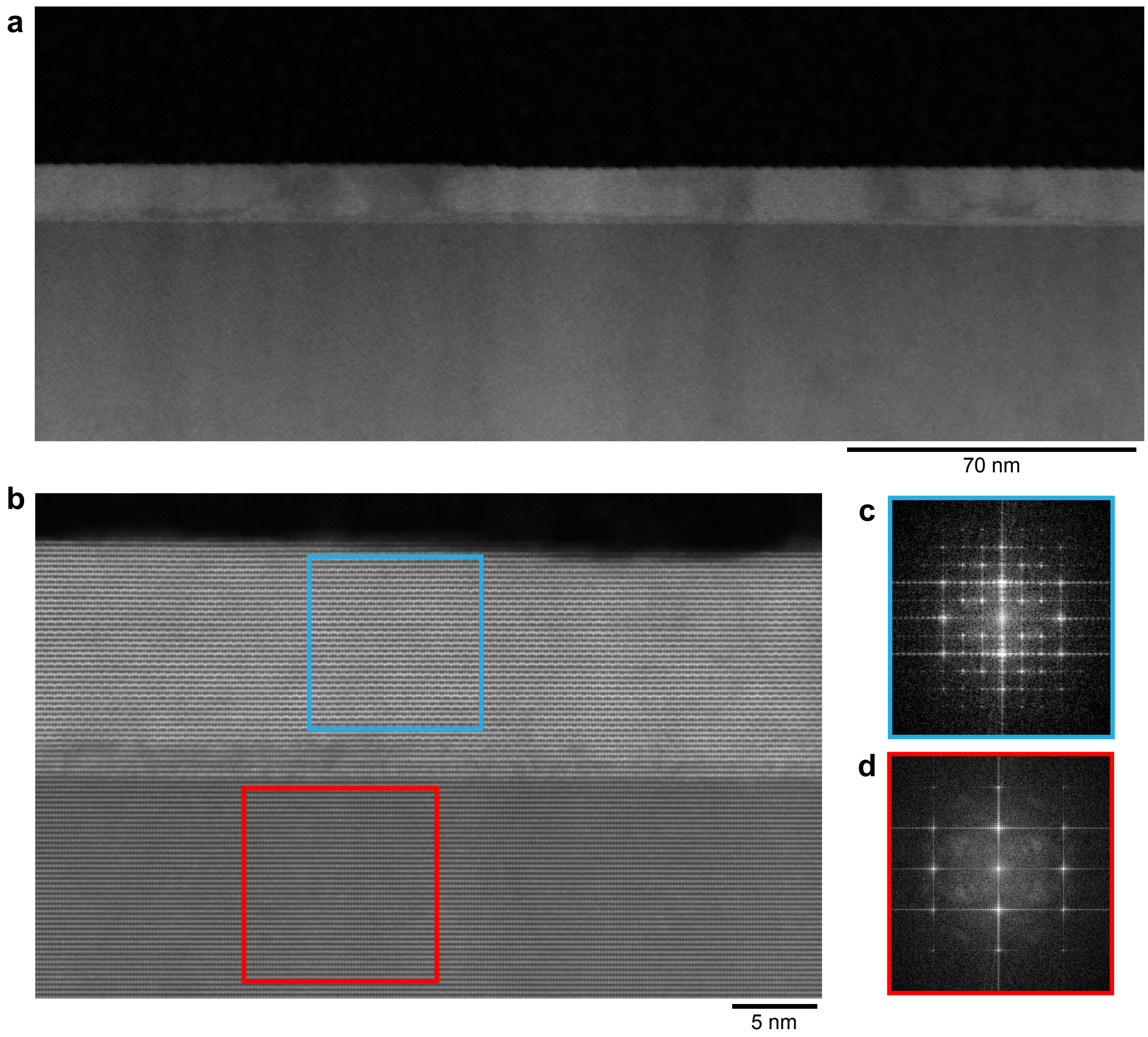

FIG. S6. HAADF-STEM imaging of a $TbInO_3$ epitaxial thin film on YSZ(111). (a) A large field-of-view HAADF-STEM micrograph of a 15 nm thick $TbInO_3$ film. (b) A higher magnification HAADF-STEM micrograph of the same film as in (a) showing the $TbInO_3$ with trimer distortion on the YSZ(111) substrate. (c) A fast fourier transform (FFT) of the trimerized region of the $TbInO_3$ film outlined in blue which shows additional peaks corresponding to the tripling of the unit cell due to the improper ferroelectric distortion. (d) FFT of the YSZ(111) substrate outlined in red which lacks the additional peaks in (c).

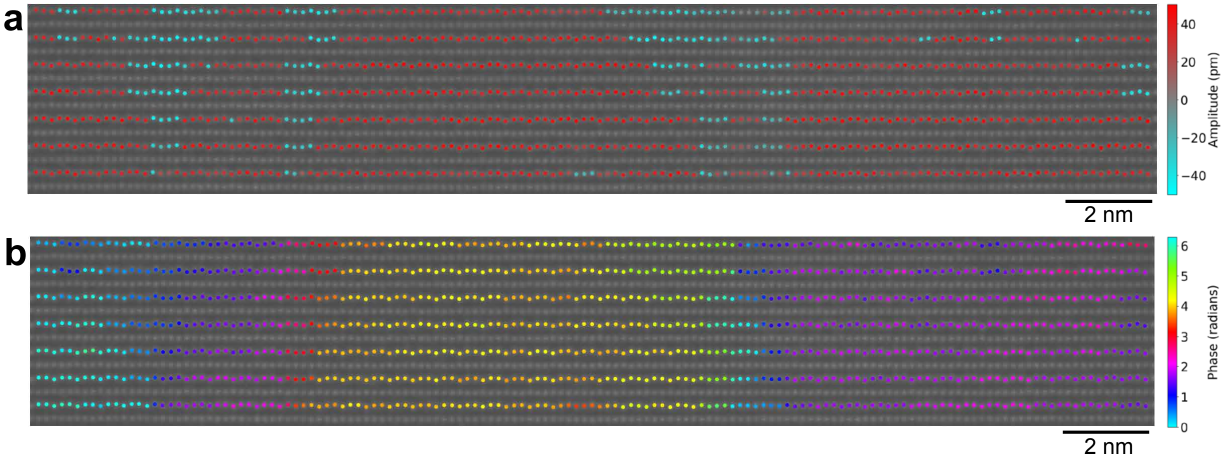

FIG. S7. Improper ferroelectric domain mapping in an 8 nm TbInO3 epitaxial thin film. (a) A map of the local distortion amplitude overlaid on a HAADF-STEM micrograph. (b) A map of the improper ferroelectric domains in the same region as (a).

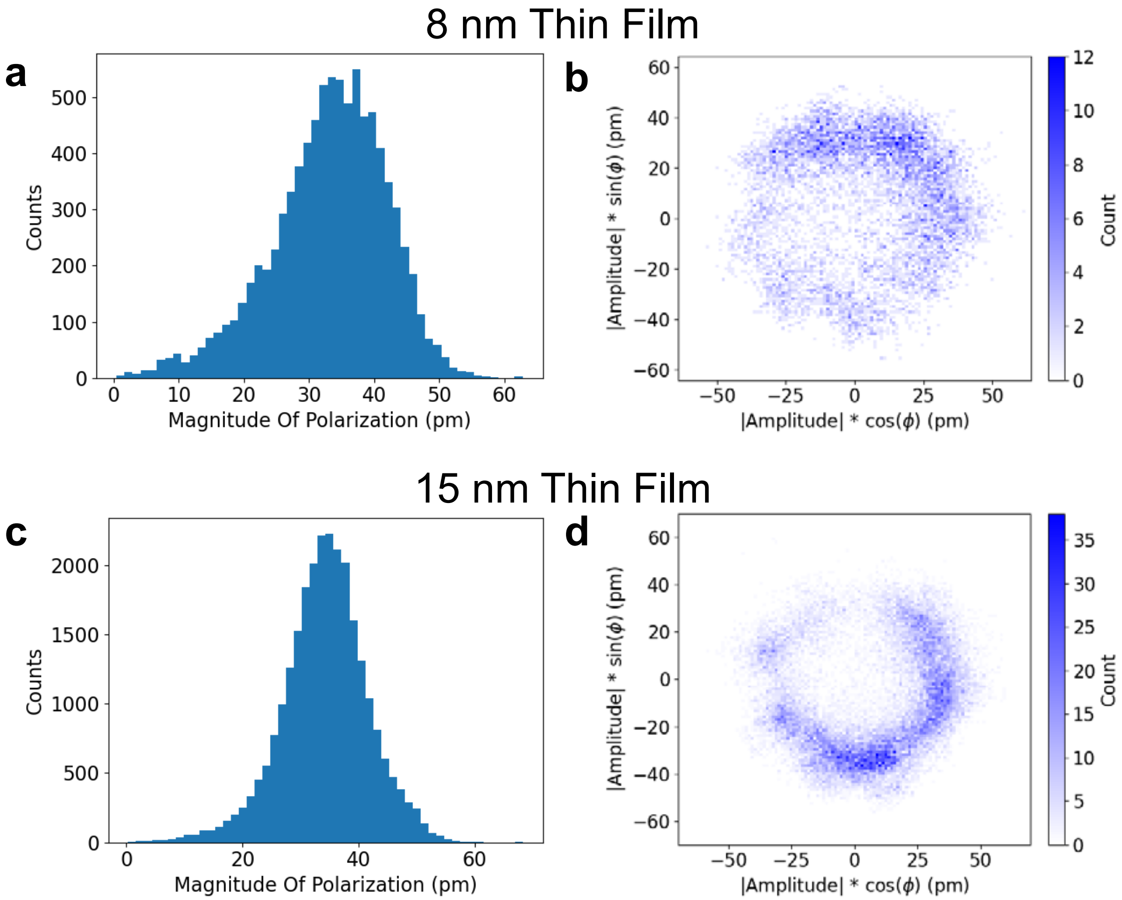

FIG. S8. Statistics of improper ferroelectric domain mapping in thin films of $TbInO_3$. (a) A histogram of local distortion magnitudes extracted from 10 unique regions of an 8 nm film with a mean of 33.06 ± 0.09 pm (N = 8810). (b) The distribution of the magnitude and phase of the polarization of the regions considered in (a). (c) A histogram of local distortion magnitudes extracted from 7 unique regions of a 15 nm film with a mean of 34.21 ± 0.05 pm (N = 26518). (d) The distribution of the magnitude and phase of the polarization of the regions considered in (c).

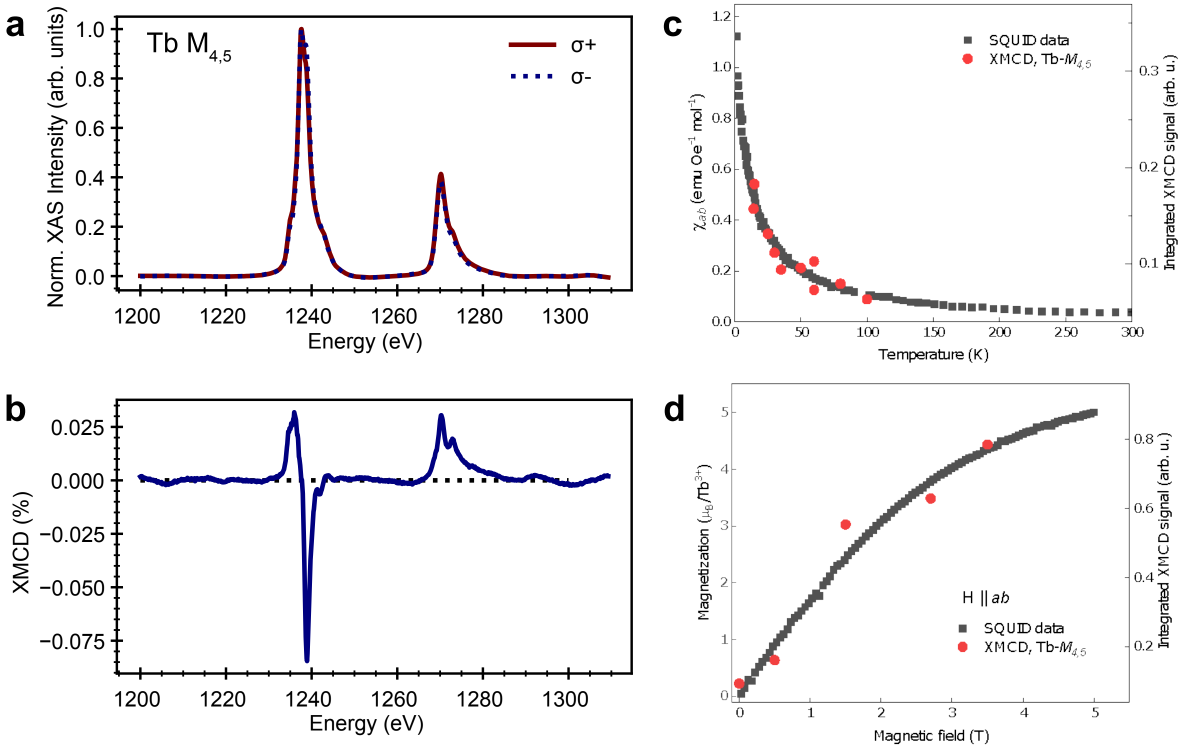

FIG. S9. X-ray magnetic Circular Dichroism (XMCD) of a $TbInO_3$ Thin Film. (a) X-ray adsorption spectroscopy of the Tb $M_{4,5}$ edge under positive (σ^+) and negative (σ^-) circularly polarized light. (b) The XMCD signal extracted from the scans in (a). (c) The correspondence between the integrated XMCD signal of a $TbInO_3$ film at different temperatures and the magnetic susceptibility measured with SQUID magnetometry. (d) The agreement between the integrated XMCD signal of a $TbInO_3$ film under various applied fields and the magnetization versus applied field measured in a SQUID magnetometer.

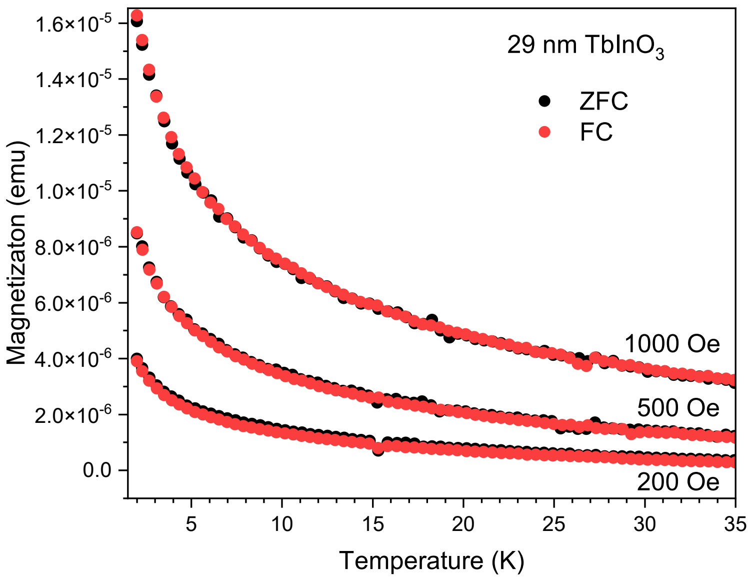

FIG. S10. Magnetization curves measured in applied fields of 1000 Oe, 500 Oe and 200 Oe along the *ab* plane under zero-field-cooled (ZFC) and field-cooled (FC) conditions. For all measurements, no splitting of the ZFC and FC curves is observed down to at least 1.8 K.

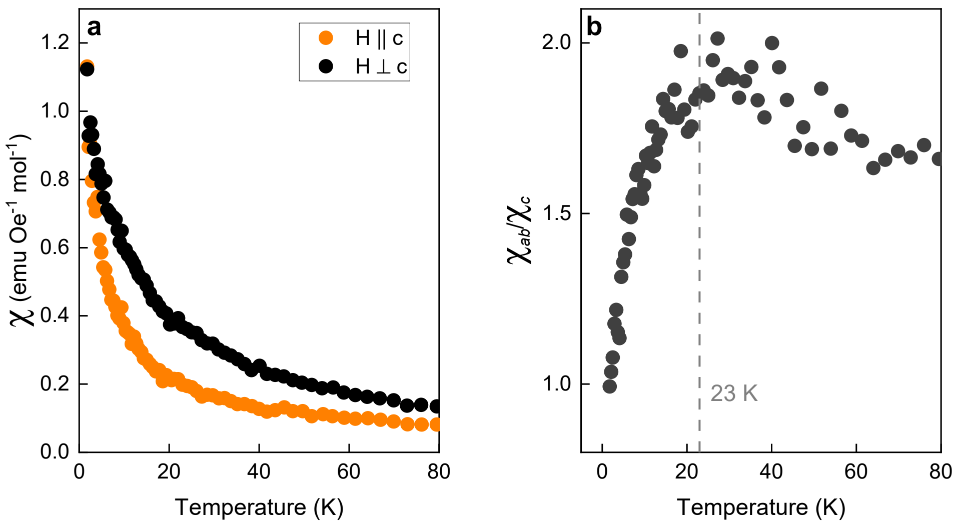

FIG. S11. Magnetic anisotropy from SQUID magnetometry (a) Temperature dependence of magnetic DC susceptibility measured with an applied field of 1000 Oe in the *ab* plane and along the *c* axis. (b) Ratio between in-plane and out-of-plane susceptibility. A reduction of the in-plane anisotropy is seen below 23 K.

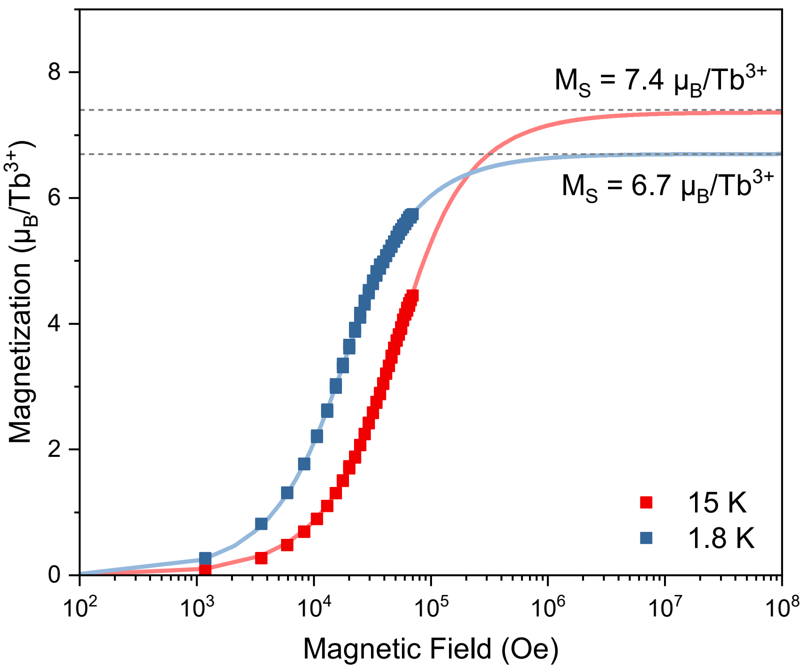

FIG. S12. Isothermal magnetization curves at 1.8 K and 15 K for a 29 nm $TbInO_3$ film yield reduced Tb^{3+} saturation moments compared to the free ion value indicated by the high temperature low-field susceptibility. Using the Langevin function as a simple model for a paramagnetic system, the saturation magnetization can be estimated from the magnetization curves. The curve measured at 15 K extrapolates to a slightly higher saturation moment than the curve measured at 1.8 K. We note however that to accurately model the magnetization behavior at low temperatures or at high magnetic field in Tb^{3+} systems, the full crystal electric field (CEF) splitting of energy levels in the ground state multiplet need to be considered. Indeed, similar behavior of reduced moment at low temperatures has been observed in other rare-earth-based oxides [1] which could be understood taking into account the temperature dependent (de-)population of CEF levels as well as the anisotropy of the system.

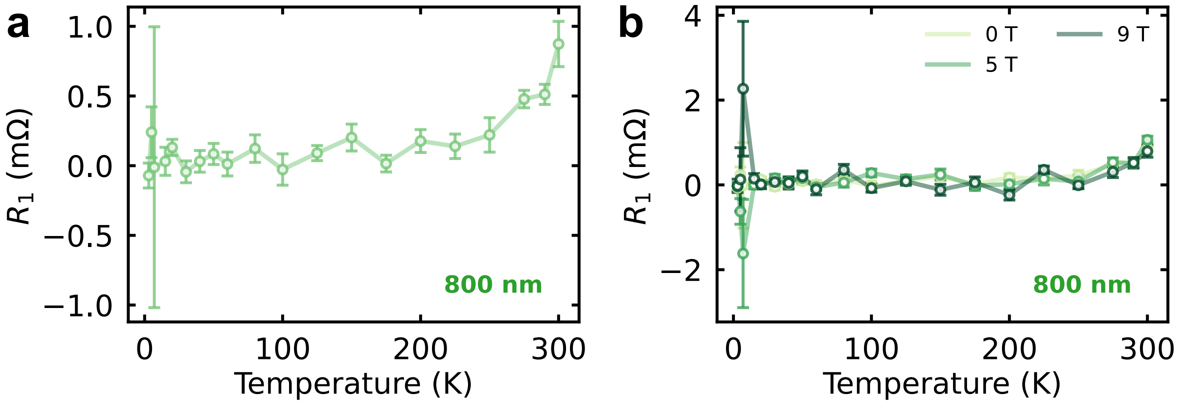

FIG. S13. Low temperature non-local transport measurements of a thin film of $TbInO_3$. (a) Non-local spin transport measurements of a device with a platinum wire spacing $d = 800\,\mathrm{K}$ from 3 to 300 K. Error bars show the least squares standard deviation fit error. (b) The same measurement on the same device in (a) but in applied fields of 0, 5, and 9 T applied in-plane and perpendicular to the length of the platinum wires.

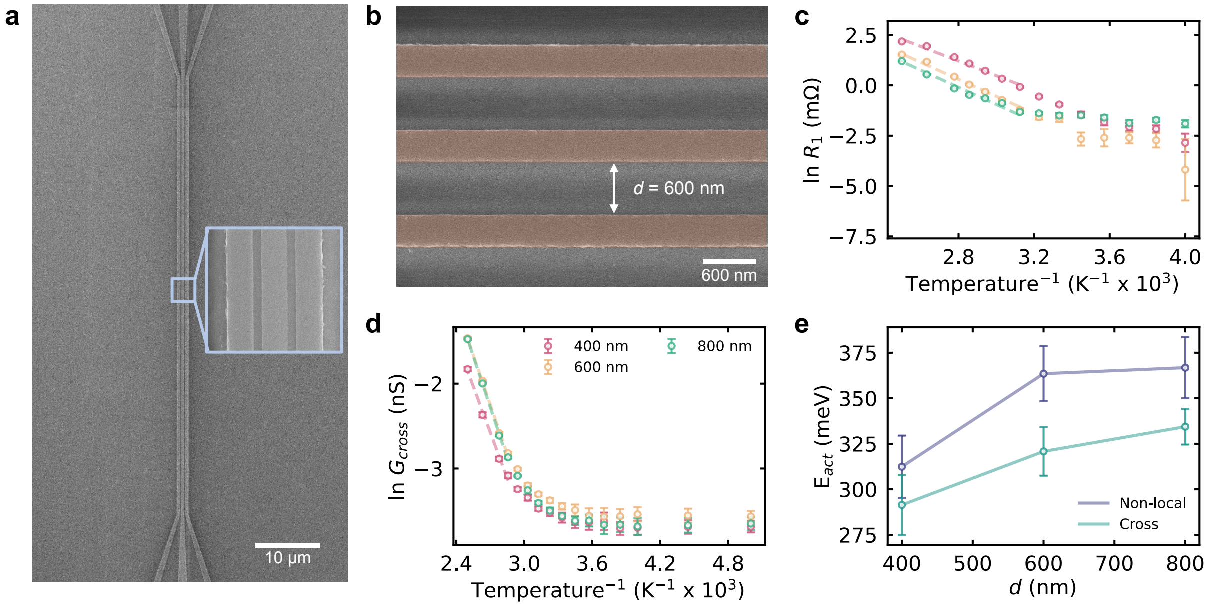

FIG. S14. Non-local transport measurements on a $TbInO_3$ thin film. (a) SEM of a non-local device with platinum wire width 500 nm and spacing d = 100 nm. (b) SEM of the non-local device, showing a spacing d of 600 nm. The orange shaded regions are the platinum wires, with a scale bar of 600 nm. (c) Natural logarithm of the symmetric R_1 signal plotted against the inverse temperature for different device spacings. The color legend is shown in panel (d). The linear region at high temperatures is fitted with a dashed straight line for extracting the activation energy. Error bars show the least squares standard deviation fit error. (d) Similar plot as in (c) but with the conductance across the device spacing. Error bars represent the standard deviation of four measurements. (e) The extracted activation energy for both the non-local signal and the signal across the device spacing at various device spacing. Error bars show the standard deviation of the least squares fit.

I. POLARIZATION CALCULATION DETAILS

Electrical polarization is calculated by multiplying Born effective charges and polar atomic displacements:

$$P_\alpha = \frac{e}{V} \sum_k Z^*_{k,\alpha\alpha} u_{k,\alpha}, \tag{1}$$

where e is the electronic charge, V is the volume of the unit cell, $Z^*_{k,\alpha\alpha}$ is the Born effective charge of ion k along direction α, and $u_{k,\alpha}$ is the polar displacement of ion k along direction α. DFT calculations described in this section are performed with the Tb f electrons kept in the core. Born effective charges are calculated using DFPT. Using our DFT-relaxed structure, we obtain a polarization of 8.14 µC cm^{-2}.

In order to calculate the polarization in the experimental film based on the measured Tb1-Tb2 distance Δ (defined in Fig. 2a of the main text), we must estimate the amplitude of the polar displacements $u_{k,\alpha}$ in the experimental structure. We note that the P6$_3cm$ crystal structure of $TbInO_3$ can be decomposed into two structural distortions: a non-polar distortion which consists of the Tb1-Tb2 displacements Δ as well as a buckling (trimerization) of the InO_5 layer which transforms like the K_3 irreducible representation of the high-symmetry P6$_3/mcm$ structure, and a polar distortion which transforms like Γ_2^-. As reported in the main text, the experimental Tb1-Tb2 distance is $\Delta_{\text{ex}} = 0.35$ Å. Analyzing our DFT-relaxed structure, we find the Tb1-Tb2 distance to be $\Delta_{\text{DFT}} = 0.45$ Å, and the full K_3 amplitude (including trimerization displacements of oxygens) to be 1.22 Å per 30-atom cell. Thus we estimate the full K_3 amplitude per cell of the experimental structure by scaling the DFT K_3 amplitude:

$$Q^{\text{ex}}_{K_3} = \frac{\Delta_{\text{ex}}}{\Delta_{\text{DFT}}} Q^{\text{DFT}}_{K_3} = 0.94 \text{ Å}. \tag{2}$$

To obtain the polar distortion amplitude that minimizes the energy given a K_3 amplitude of 0.94 Å, we create a series of structures with $Q_{K_3} = 0.94$ Å and polar distortion amplitudes $Q_{\Gamma_2^-}$ varying between 0 and 0.6 Å. We use DFT to calculate the total energy of each of these structures; the result is shown in Fig. S15. The minimum of this energy surface then provides an estimate of the experimental polar distortion amplitude, which is $Q_{\Gamma_2^-} = 0.25$ Å. Finally, we use DFPT to compute Born effective charges of the $Q_{K_3} = 0.94$ Å, $Q_{\Gamma_2^-} = 0.25$ Å structure and use Eq. 1 to compute the experimental polarization value of 7.11 µC/cm^2.

We also show the calculated band structure and density of states of $TbInO_3$ in Fig. S16.

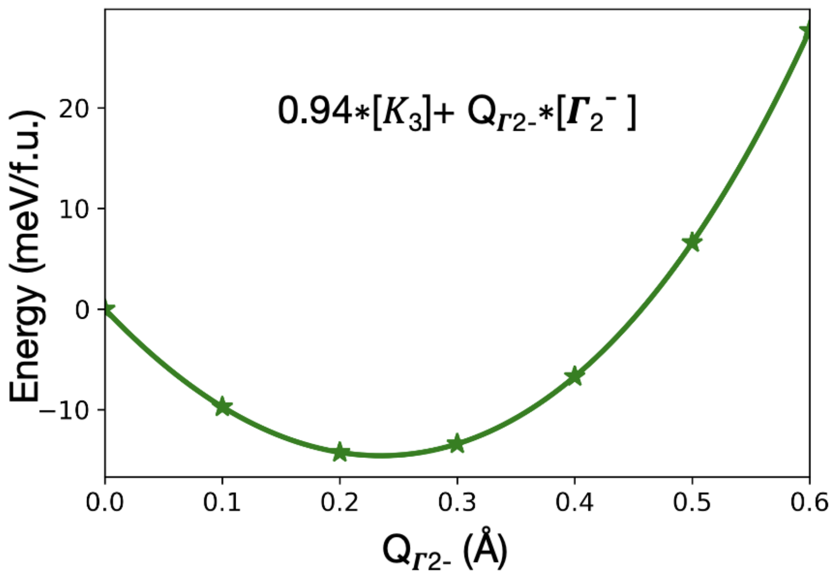

FIG. S15. DFT total energy versus $Q_{\Gamma_2^-}$ polar distortion amplitude.

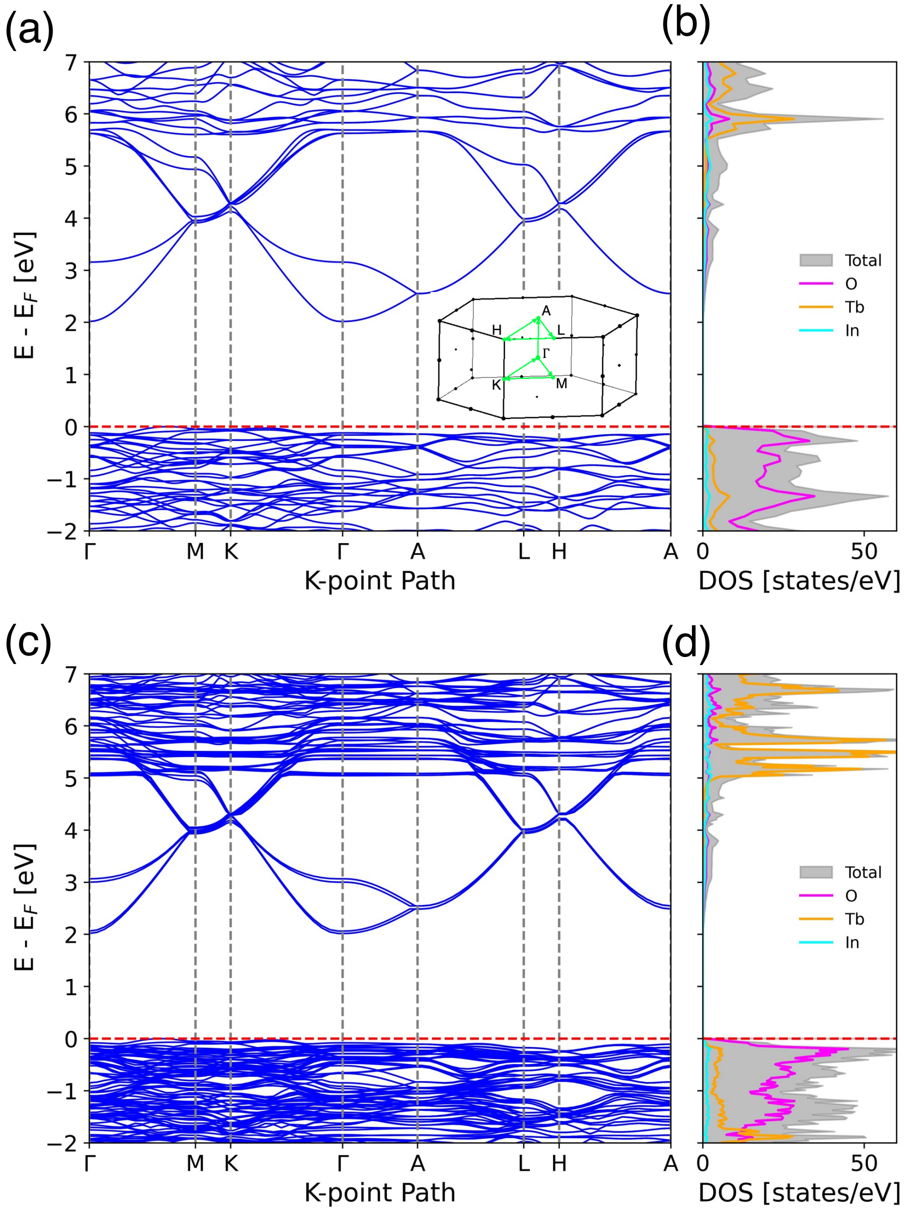

FIG. S16. Bandstructure and density of states (DOS) of $TbInO_3$ calculated with (a-b) DFT and (c-d) DFT + U + spin-orbit coupling (SOC). The bandgap in both calculations is 2.02 eV, in good agreement with the experimental value of 2.2 eV [2]. The localized Tb-f states in the DFT+ U + SOC calculation (c-d) are high above the Fermi level (> 5 eV). The lattice parameters calculated by structural relaxation with DFT are a=6.373, c=12.382 Å, and those calculated with DFT+ U + SOC are a=6.333, c=12.289 Å. These values are within a few percent of the experimental lattice parameters, which are a=6.32, c=12.31 Å[3].

II. DC MAGNETIZATION MEASUREMENTS

The subtraction of the substrate induced magnetic background was performed by directly subtracting the fitted magnetization values (given by the amplitude of the raw waveform recorded in the DC scan mode) for each measurement point (T, H). To account for slight differences in the temperature independent diamagnetic background due to small variations in sample mounting, the diamagnetic background was measured at room temperature and first subtracted from all data points. We note that it is alternatively also possible to subtract the background by subtracting the measured waveforms acquired in the DC scan mode at each measurement point (T, H) and then fit the difference signal to extract the magnetization value. This is especially important in the case of a non-uniform background from the sample holder, which cannot be well modeled as a point source, in combination with small sample signals. In that case, the fitting of the individual waveforms will not be reliable, and hence the method of directly subtracting the fitted magnetization values would not be appropriate. In our case, however, since the background signal being subtracted is originating in the substrate, which has the same size and shape as the substrate+film sample, the two methods yield equivalent results within the error of our measurements. We compare the two methods in Fig. S17. To fit the raw waveforms, we use the same fitting function as used by the system (Quantum Design, MPMS3). As can be seen, the extracted magnetic moments fall well within 5% for all measurements except in the case where either (i) one sample exhibits zero magnetization (because the diamagnetic and paramagnetic contributions exactly cancel), as can be seen in Fig. S17(b) or (ii) the film-only contribution is near zero (Fig. S17(d)). In these cases, we observe an increased discrepancy (up to 15%) between the subtracted fitted magnetization values compared to the fit of the subtracted raw waveforms.

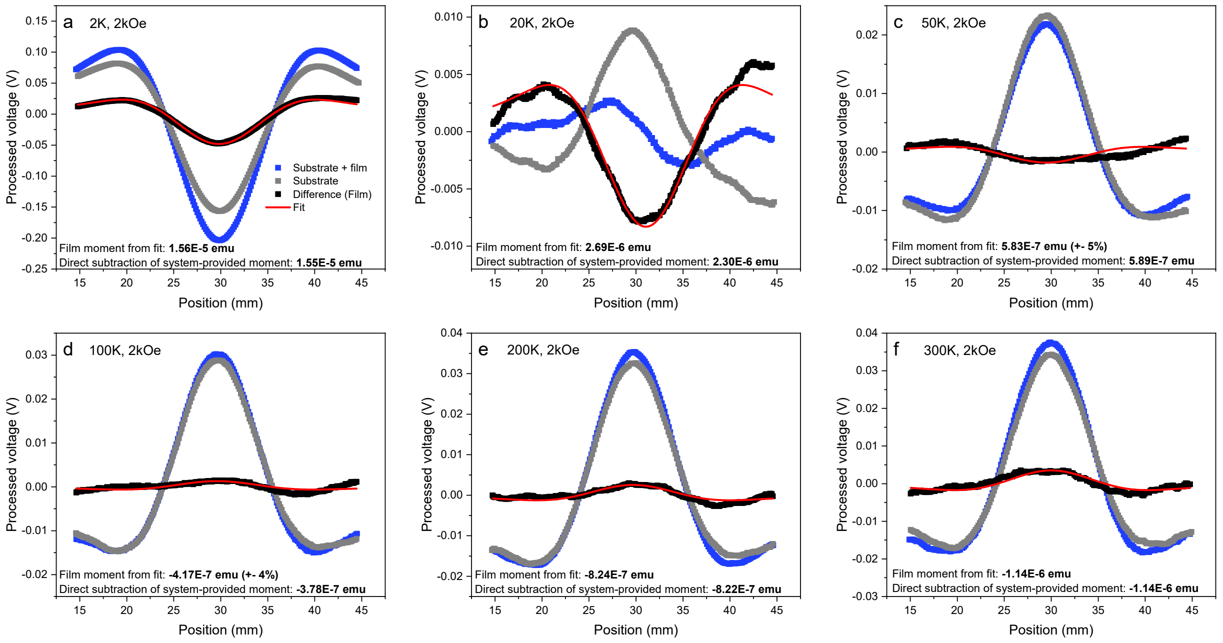

FIG. S17. Comparison of the raw SQUID waveforms measured on a $TbInO_3$ film on YSZ and on the bare substrate with an applied field of 2000 Oe along the *ab* plane at the following temperatures: (a) 2 K, (b) 20 K, (c) 50 K (d) 100 K (e) 200 K and (f) 300 K.

III. SCANNING SQUID SUSCEPTIBILITY MEASUREMENT DETAILS

The YSZ substrate has a paramagnetic susceptibility due to trace amounts of impurities. The impurities are presumably dilute and non-interacting, thus the substrate does not order and we find the Curie-Weiss temperature of the substrate to be $T_{\mathrm{C}} \sim 0\,\mathrm{K}$ (see below). Since the susceptibility of the substrate grows as $T \to 0$, and the volume of the substrate is orders of magnitude larger than the thin film, care must be taken to disentangle the substrate contribution to the susceptibility measured from the thin film on the substrate. In addition, we find there is significant variation in the paramagnetic susceptibility from substrate to substrate, making it necessary to measure the susceptibility of the substrate that the thin film sample is grown on rather than using a bare substrate as a reference. To address these concerns, we use ion-milling to etch away part of the 38 nm $TbInO_3$ film revealing the bare YSZ substrate (Fig. S18). We then use a scanning SQUID setup to locally probe the susceptibility of the bare substrate and the film combined with the substrate side-by-side to determine the substrate contribution.

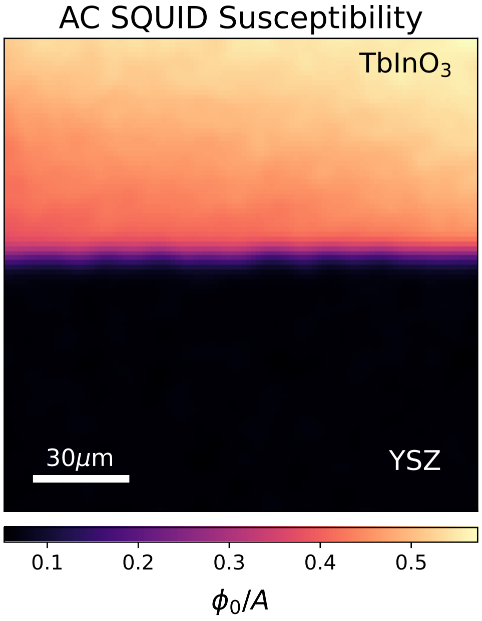

FIG. S18. Spatial image of the AC SQUID susceptibility (corresponding to ΔM as described in Sec. III) taken at the boundary of the $TbInO_3$ film and where it has been ion-mill etched away revealing the bare YSZ substrate underneath. The scanning SQUID has μm scale spatial resolution, and the $TbInO_3$ appears uniform on these length scales.

The SQUID sensor features two coils: the inner pick-up coil with an inner-radius of 0.75 µm and an outer-radius of 1.6 µm, and the outer field coil with an inner-raduis of 3 µm and an outer-radius of 6 µm. The pick-up coil is part of the SQUID such that we can detect magnetic flux threading it. To measure magnetic susceptibility, we flow an ac current through the field coil, which applies a local magnetic field to the sample [4]. Far from the sample surface, we measure the mutual inductance between these two coils M_0 (measured to be 384.6 ϕ_0/A for the SQUID sensor used here). When we approach the SQUID to the sample surface (Fig. S19(a) and (c)), the sample responds to the local field produced by the field-coil such that the amount of flux in the pick-up coil changes. We measure this as a change in the mutual inductance ΔM.

The change in mutual inductance with SQUID-sample distance can be modeled for bulk and thin film paramagnets [5]:

$$\frac{\Delta M_{\text{bulk}}}{M_0} = \frac{\chi}{\chi+2}\frac{1}{(1+4(\frac{z}{a})^2)^{3/2}}, \tag{3}$$

$$\frac{\Delta M_{\text{film}}}{M_0} = \frac{\chi^2+2\chi}{\chi+1}\frac{3tz}{a^2(1+4(\frac{z}{a})^2)^{5/2}}. \tag{4}$$

Here χ is the volume susceptibility, a is the effective field-coil radius (here estimated to be 3.26 µm), t is the film thickness, and z is the SQUID-sample distance. We take approach curves, i.e. we measure the response as a function of z, with the SQUID on both the bare substrate and the sample + substrate as a function of temperature and frequency of the AC current flowing through the field-coil. We fit the data for the bare substrate, ΔM_{sub}, with the expression for a bulk paramagnet, and the data collected on the combined film and substrate, ΔM_{sa}, with a sum of the expression for a thin paramagnetic film and substrate contributions. These expressions are given by:

$$\frac{\Delta M_{\text{sub}}(z,T)}{M_0} = \frac{\Delta M_{\text{bulk}}(z,T)}{M_0} = \frac{\chi_{\text{sub}}(T)}{\chi_{\text{sub}}(T)+2}\frac{1}{(1+4(\frac{z}{a})^2)^{3/2}}, \tag{5}$$

and

$$\begin{aligned}\frac{\Delta M_{\text{sa}}(z,T)}{M_0} &= \frac{\Delta M_{\text{film}}(z,T)}{M_0} + \frac{\Delta M_{\text{sub}}(z,T)}{M_0}\\ &= \frac{\chi_{\text{film}}(T)^2+2\chi_{\text{film}}(T)}{\chi_{\text{film}}(T)+1}\frac{3tz}{a^2(1+4(\frac{z}{a})^2)^{5/2}} + \frac{\Delta M_{\text{sub}}(z,T)}{M_0}.\end{aligned} \tag{6}$$

To constrain the fitting, we fit all approach curves taken on both the bare substrate and the sample at different temperatures and frequencies in a single minimization procedure. Examples of approach curves at a few temperatures collected at 7 Hz and their fits are shown for the bare substrate in Fig. S19(a) and for the film and substrate in Fig. S19(c).

In this fitting procedure, we constrain $\chi_{\mathrm{sub}}(T)$ to follow Curie-Weiss behavior $\chi_{\mathrm{sub}}(T) = \frac{C}{T-T_c}$. For a small $\chi_{\mathrm{sub}}(T)$ and a fixed SQUID-sample distance $z = z^*$, $\Delta M_{\mathrm{sub}}(z^*, T) \propto \chi_{\mathrm{sub}}(T)$, i.e. the temperature-dependence of $\chi_{\mathrm{sub}}(T)$ can be captured at a constant height without knowing details about the geometry. In Fig. S19(b), we show that the measured $\Delta M_{\mathrm{sub}}^{-1}(z^*, T)$ at a fixed height z^* is linear in T, justifying the constraint on $\chi_{\mathrm{sub}}(T)$ to follow the Curie-Weiss behavior. Additionally we plot $\Delta M_{\mathrm{sub}}^{-1}(z^*, T)$ obtained from fitting showing good agreement with the measured substrate data and linear in T scaling due to the constraint on $\chi_{\mathrm{sub}}(T)$.

In Fig. S19(d), we plot the measured sample signal (film + substrate) at a fixed SQUID-sample distance $z = z^*$, $\Delta M_{\mathrm{sa}}(z^*, T)$ along with the contribution from only the $TbInO_3$ film obtained from the fitting procedure $\Delta M_{\mathrm{film}}(z^*, T)$. The difference between ΔM_{sa} and ΔM_{film} in Fig. S19(d) is a direct indication of the substrate contribution to the susceptibility. Due to the Curie-Weiss behavior of the substrate contribution, it becomes most apparent at the lowest temperatures, and causes the raw sample data to turn upward. We emphasize that the turnover at $T \sim 1$ K is present in the raw data, and is not an artifact from fitting or accounting for the substrate contribution. Additionally, we note that any paramagnetic signal from the substrate would tend to increase the measured ΔM_{sa}, thus the turnover is not attributable to the substrate. We measured another sample over a more limited temperature range (∼50 - 900 mK) and without removing part of the film to reveal the bare substrate. Although we could not reliably disentangle the substrate contribution in this measurement, nor see a turnover in susceptibility in the more limited temperature range, we did observe a decreasing susceptibility (film+substrate) with decreasing temperature in the range ∼300 - 900 mK (the substrate paramagnetism begins to dominate at $T <\sim 300$ mK). This is qualitatively consistent with the turnover seen here. We also note that although there is some uncertainty in the exact value of $\chi(T)$, in the limit that χ is small, $\Delta M \propto \chi$ and all geometric factors cancel when looking at the variation of χ with temperature. Thus the $\chi(T)$ curve may be scaled with a corrective factor, but the shape of $\chi(T)$ is robust against the exact fit parameters. The susceptibility χ plotted in the main text Fig. 3(d)

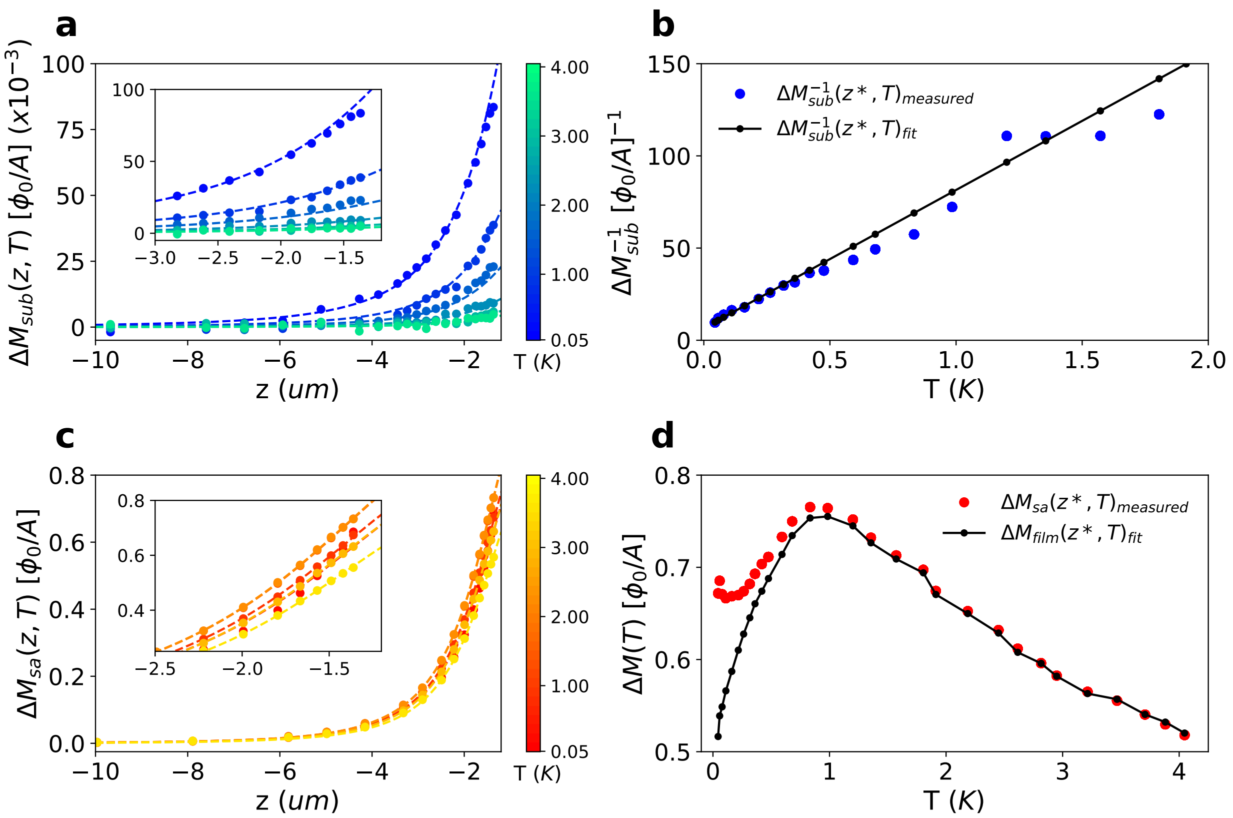

FIG. S19. (a) SQUID approach curves measured on the bare substrate along with their fits at a few select temperatures and acquired using 7 Hz. (b) At a fixed $z = z^*$ (line cut in (a)), and for small signals $\Delta M^{-1}_{\mathrm{sub}}(T) \propto \chi^{-1}_{\mathrm{sub}}(T)$. The measured data (blue) shows a linear dependence in T, indicating Curie-Weiss behavior. When fitting the substrate and sample approach curves together, $\chi_{\mathrm{sub}}(T)$ is constrained to follow a Curie-Weiss behavior as shown by the same line cut taken at $z = z^*$ for the fit curves of the substrate (black) with $T_c = -85$ mK. (c) Approach curves taken on the sample (film+substrate) along with their corresponding fits at select temperatures (at 7 Hz). (d) $\Delta M(T)$ plotted at a fixed $z = z^*$ (line cut in (c)) for the measured sample (film+substrate) signal (red) and the corresponding signal from just the $TbInO_3$ film after factoring out the substrate contribution (black). The signal from only the $TbInO_3$ film corresponds to $\chi(T)$ reported in the main text.

is obtained by converting ΔM_{film} from Fig. S19(d) to the volume susceptibility χ_{film} via Eq. 6. The molar susceptibility (in emu/mol) is found from the volume susceptibility by $\chi = M\chi_{\mathrm{film}}/4\pi\rho$ where M and ρ are the molar mass (321.75 g/mol) and the density (7.93 g cm^{-3}) of $TbInO_3$.

IV. ANALYSIS OF AC SUSCEPTIBILITY

In this section we provide an analysis of the frequency dependence of the AC susceptibility data presented in Fig. 3(d) in the main text. In many experimental manifestations of a QSL state, the spins gradually lose their ability to reorient freely at the lowest temperatures; instead, they only rotate over much longer timescales that increase with decreasing temperature. Measurements on the bulk crystal $TbInO_3$ show that although the spin fluctuations slow down with decreasing temperature, the spins remain in a fluctuating state down to the lowest temperatures (100 mK) measured by µSR [3]. In the case where spin freezing occurs, it is typically accompanied by a broad turnover in the AC susceptibility as a function of temperature, and the temperature at which the AC susceptibility has a maximum is often referred to as the freezing temperature. This freezing temperature is frequency dependent, see e.g. [6–8], and roughly occurs when the experimental frequency becomes comparable to the characteristic relaxation rates in the system.

We observe a frequency dependence of the AC susceptibility and the temperature at which the turnover occurs. In Fig. S20(a) we analyze the temperature dependence of the freezing temperature by fitting a polynomial to the AC susceptibility to interpolate between the data points and extract the position of the maximum, T_m, as a function of frequency shown in Fig. S20(b). Following Refs. [6, 7, 9], we fit the frequency dependence of T_m to a power law expression:

$$\tau = \tau^* \left(\frac{T_m}{T_g} - 1 \right)^{-z\nu} \tag{7}$$

where $\tau = (2\pi f)^{-1}$, τ^* is the characteristic relaxation time, T_g is the static spin glass freezing temperature, and $z\nu$ is the critical exponent. The fit to our extracted T_m is shown in Fig. S20(c) and shows good agreement with a power law scaling of T_m. We find the fit parameters to be: $\tau^* \sim 2.0{\times}10^{-5}$ s, $T_g \sim 0.63$ K, and $z\nu \sim 11.3$. An additional analysis [6, 7, 9, 10] is extracting the temperature shift of the freezing temperature ΔT_m per decade change in frequency:

$$K = \frac{1}{T_m} \frac{\Delta T_m}{\Delta log_{10}\omega} \tag{8}$$

We extract K for our data by fitting to Eq. 8 as shown in Fig. S20(d) and find a value of K $\sim$ 0.076. The values of τ^* and K obtained from our analysis are larger than than those

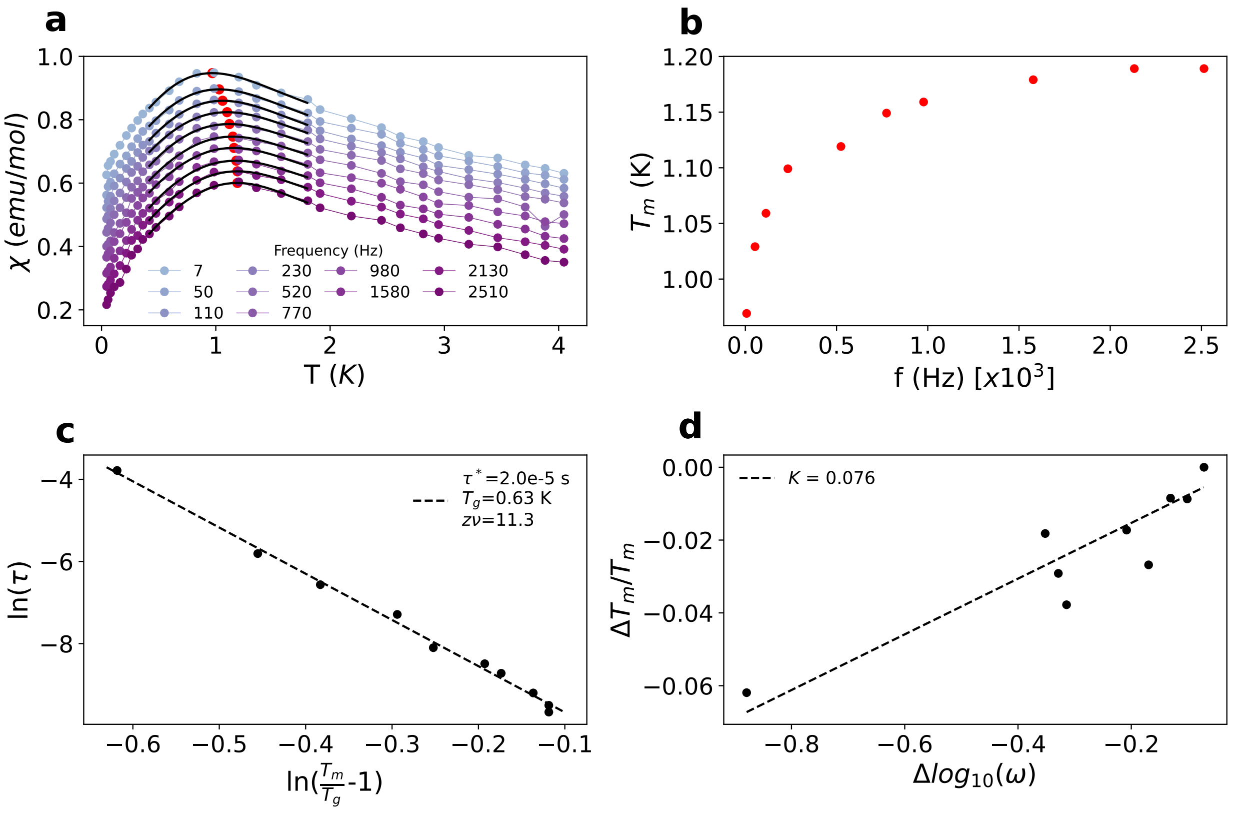

FIG. S20. (a) AC susceptibility as a function of temperature and frequency. The data at each frequency are fit to a polynomial near the turnover (black lines) and the temperature T_m is determined as the temperature at which the polynomial function has a maximum (red points). The y-axis corresponds to the 7 Hz dataset, all other curves have been offset for clarity. (b) Frequency dependence of the freezing temperature T_m, corresponding to the red points in (a). (c) Frequency dependence of the freezing temperature with the line representing the best power law fit to Eq. 7. (d) Temperature shift of the freezing temperature as a function of changes in frequency. The line corresponds to the best fit to Eq. 8.

of a canonical spin glass: $\tau^* \sim 10^{-12} - 10^{-13}$ and $K \sim 10^{-3}$. Our parameters fall closer to, although do not coincide with, those expected for a cluster spin glass ($\tau^* \sim 10^{-7} - 10^{-10}$ and $K \sim 10^{-2}$) in which local groups of spins are ordered into domains, but each domain fluctuates in a similar way to individual spins in a spin glass [8–11]. We note, however, that the extracted parameters of τ^* and K are sensitive to the extraction method for T_m e.g. the order and temperature range of the polynomial fit, so precise classification of our $TbInO_3$ sample through considering the values of τ^* and K is fairly speculative.

Spin freezing does occur in geometrically frustrated magnetic systems, but in principle some disorder in the antiferromagnetic exchange needs to be present. While the thin film shows a high level of crystallinity, a possible source of disorder in addition to defects can be ferroelectric domains. We note that a downturn in the AC susceptibility has been recently observed in bulk crystals of the isostructural hexagonal rare-earth indate $DyInO_3$ [12], and has been interpreted as a signature of spin freezing with ferroelectric domains as a possible source for disorder. Taken together, our measurements suggest that spin freezing occurs at low temperature, however, given the many different mechanisms that can lead to a downturn in the AC susceptibility, it is challenging to truly pinpoint its origin.

REFERENCES

[1] M. Gingras *et al.*, Thermodynamic and single-ion properties of Tb^{3+} within the collective paramagnetic-spin liquid state of the frustrated pyrochlore antiferromagnet $Tb_2Ti_2O_7$, Physical Review B **62**, 6496 (2000).

[2] P. Chen *et al.*, Magnetochromic effect in multiferroic $RIn_{1-x}Mn_xO_3$ (R= Tb, Dy), Physical Review B **91**, 205130 (2015).

[3] L. Clark *et al.*, Two-dimensional spin liquid behaviour in the triangular-honeycomb antiferromagnet $TbInO_3$, Nature Physics **15**, 262 (2019).

[4] J. Kirtley *et al.*, Scanning squid susceptometers with sub-micron spatial resolution, Review of Scientific Instruments **87**, 093702 (2016).

[5] J. Kirtley *et al.*, Scanning squid susceptometry of a paramagnetic superconductor, Physical Review B **85**, 224518 (2012).

[6] K. Binder and A. P. Young, Spin glasses: Experimental facts, theoretical concepts, and open questions, Reviews of Modern Physics **58**, 801 (1986).

[7] P. Bag, P. Baral, and R. Nath, Cluster spin-glass behavior and memory effect in $Cr_{0.5}Fe_{0.5}Ga$, Physical Review B **98**, 144436 (2018).

[8] M. Roy-Chowdhury, M. S. Seehra, and S. Thota, Optimized analysis of the AC magnetic susceptibility data in several spin-glass systems using the Vogel–Fulcher and Power laws, AIP Advances **13**, 115020 (2023).

[9] Z. Fu *et al.*, Coexistence of magnetic order and spin-glass-like phase in the pyrochlore antiferromagnet $Na_3Co(CO_3)_2Cl$, Physical Review B **87**, 214406 (2013).

[10] J. A. Mydosh, Spin glasses: redux: an updated experimental/materials survey, Reports on Progress in Physics **78**, 052501 (2015).

[11] B. Antic *et al.*, Spin glass formation in Li-substituted Co_2TiO_4 spinel, Journal of Physics: Condensed Matter **16**, 651 (2004).

[12] X. Xu, C. Won, and S.-W. Cheong, Frustrated magnetism and ferroelectricity in a Dy^{3+}-based triangular lattice, Crystals **13**, 971 (2023).
